# Effect of caffeine on vestibular evoked myogenic potential: a systematic review with meta-analysis^☆^

**DOI:** 10.1016/j.bjorl.2017.11.003

**Published:** 2017-12-24

**Authors:** Maria Eduarda Di Cavalcanti Alves de Souza, Klinger Vagner Teixeira da Costa, Pedro de Lemos Menezes

**Affiliations:** a Universidade Federal de Alagoas (UFAL), Rede Nordeste de Biotecnologia (RENORBIO), Biotecnologia em Saúde, Maceió, AL, Brazil; b Universidade Estadual de Ciências da Saúde de Alagoas (UNCISAL), Maceió, AL, Brazil

**Keywords:** Vestibular function tests, Coffee, Evoked motor potential, Vestibular nerve, Testes de função vestibular, Café, Potencial evocado motor, Nervo vestibular

## Abstract

**Introduction:**

Caffeine can be considered the most consumed drug by adults worldwide, and can be found in several foods, such as chocolate, coffee, tea, soda and others. Overall, caffeine in moderate doses, results in increased physical and intellectual productivity, increases the capacity of concentration and reduces the time of reaction to sensory stimuli. On the other hand, high doses can cause noticeable signs of mental confusion and error induction in intellectual tasks, anxiety, restlessness, muscle tremors, tachycardia, labyrinthine changes, and tinnitus.

**Objective:**

Considering that the vestibular evoked myogenic potential is a clinical test that evaluates the muscular response of high intensity auditory stimulation, the present systematic review aimed to analyze the effects of caffeine on vestibular evoked myogenic potential.

**Methods:**

This study consisted of the search of the following databases: MEDLINE, CENTRAL, ScienceDirect, Scopus, Web of Science, LILACS, SciELO and ClinicalTrials.gov. Additionally, the gray literature was also searched. The search strategy included terms related to intervention (caffeine or coffee consumption) and the primary outcome (vestibular evoked myogenic potential).

**Results:**

Based on the 253 potentially relevant articles identified through the database search, only two full-text publications were retrieved for further evaluation, which were maintained for qualitative analysis.

**Conclusion:**

Analyzing the articles found, caffeine has no effect on vestibular evoked myogenic potential in normal individuals.

## Resumo

**Introdução:**

A cafeína pode ser considerada a droga mais consumida por adultos em nível mundial, podendo ser encontrada em inúmeros alimentos, como no chocolate, café, chá, refrigerante e outros. Em geral, a cafeína em doses moderadas, produz ótimos rendimentos físico e intelectual, aumenta a capacidade de concentração e diminui o tempo de reação aos estímulos sensoriais. Por outro lado, doses elevadas podem causar sinais perceptíveis de confusão mental e indução de erros em tarefas intelectuais, ansiedade, nervosismo, tremores musculares, taquicardia, alterações labirínticas e zumbido.

**Objetivo:**

Considerando que o potencial evocado miogênico vestibular é um teste clínico que avalia a resposta muscular decorrente de estimulação auditiva de alta intensidade, a presente revisão sistemática objetivou analisar o efeito da cafeína sobre o potencial evocado miogênico vestibular.

**Método:**

A formulação deste trabalho consistiu na busca dos estudos nas seguintes bases de dados: MEDLINE, CENTRAL, *ScienceDirect*, *Scopus*, *Web of Science*, LILACS, SciELO e ClinicalTrials.gov. Adicionalmente, a literatura cinzenta também foi pesquisada. A estratégia de busca incluiu termos relacionados à intervenção (consumo de cafeína ou café) e ao desfecho primário (potencial evocado miogênico vestibular).

**Resultados:**

A partir de 253 registros potencialmente relevantes identificados através da busca nas bases de dados, apenas duas publicações em texto completo foram recuperadas para avaliação mais aprofundada, sendo estas mantidas para a análise qualitativa.

**Conclusão:**

Diante dos artigos encontrados a cafeína não tem efeito sobre o potencial evocado miogênico vestibular em sujeitos normais.

## Introduction

Caffeine can be considered the most consumed drug by adults worldwide, and can be found in several foods, such as chocolate, coffee, tea, soda and others.^1^ It is also present in many supplements, diuretics, weight loss products and alertness maintenance products.^2^ In addition to all these uses, caffeine is often used prior to performing physical exercises, aiming to delay fatigue and improve physical performance.^3^, ^4^, ^5^

Until the early 1990s, there were few review studies available in the literature that indicated the possible ergogenic effects of caffeine (an effect that increases the capacity for bodily or mental work, especially by eliminating fatigue symptoms, aiming at improving performance).^4^, ^6^, ^7^ Therefore, it was only after a few years that great importance started to be given to the study of caffeine as a possible ergogenic resource, which contributed to a greater production of studies in this area.^5^, ^8^, ^9^, ^10^, ^11^

After coffee intake, it is estimated that caffeine takes 30–45 min to reach its peak plasma concentration,^12^ with a plasma half-life of approximately 3–7 h.^3^ Its action can reach all tissues, because it is transported by the bloodstream, later being metabolized by the liver and excreted by the urine as its co-products.^5^, ^8^

It is believed that caffeine has central and peripheral mechanisms of action that can trigger important metabolic and physiological changes, which could improve athletic performance.^5^, ^13^, ^14^, ^15^, ^16^ Regarding the neurophysiological aspects, caffeine acts as a stimulant, increasing the central nervous system activity by blocking adenosine receptors in brain and spinal cord neurons. At the same time, the adenosine bound to these receptors results in a calming effect, which is determined by the administered dose and the individualized metabolism.^17^, ^18^

In general, caffeine in moderate doses (200–300 mg), results in increased physical and intellectual productivity, enhances concentration capacity and reduces the time of reaction to sensory stimuli.^19^, ^20^ On the other hand, high doses (>600 mg/day) can cause noticeable signs of mental confusion and error induction in intellectual tasks, anxiety, restlessness, muscle tremors, tachycardia, labyrinthine changes, and tinnitus.^20^, ^21^, ^22^

Considering that the Vestibular Evoked Myogenic Potential (VEMP) is a clinical test that evaluates the muscle response caused by high-intensity auditory stimulation used to evaluate vestibular function through the muscle reflex response and that caffeine can alter the mechanisms of this potential in several ways, the present systematic review aimed to analyze the effect of caffeine on VEMP.

## Methods

The construction of this systematic review aimed to answer the following question: What is the effect of caffeine on vestibular evoked myogenic potential? Based on this question, the present is reported according to the Preferred Reporting Items for Systematic Reviews and Meta-Analyses (PRISMA) Statement.^23^ The protocol was previously published in the PROSPERO database (http://www.crd.york.ac.uk/PROSPERO), under number CRD42017068051.

Considering the adherence to the PRISMA criteria, the issues addressed in the objective refer to the first three elements of the PICO strategy, with healthy adult patients, intervention using caffeine, comparison with normal adults who did not receive caffeine, and the outcome being vestibular evoked myogenic potential variations.

### Search strategy

The following databases were searched up to May 2017: MEDLINE, CENTRAL, ScienceDirect, Scopus, Web of Science, LILACS, SciELO and ClinicalTrials.gov. Additionally, the gray literature databases were also searched: OpenGrey.eu, DissOnline.de, NYAM.org and ClinicalEvidence.com. There was no manual search of the included articles and experts in the area were not contacted to avoid the bias risk.^24^

The search strategy included terms related to the intervention (caffeine or coffee consumption) and the primary outcome (vestibular evoked myogenic potential). These descriptors were used in English to search most of the databases; however, articles were required to have at least the title and/or abstract in English to be part of the present selection. The complete search strategy is shown in Appendix 1. The search was not restricted to any year of publication or language.

### Eligibility criteria

This review included clinical trials or observational studies that met the following criteria: (1) presence of a group with caffeine intake (coffee, tea, chocolate or cola drinks) and a control group; (2) and the evaluation of VEMP. There were no restrictions based on gender, ethnicity or comorbidities. As a minimum requirement, the studies must have assessed VEMP as a result and reported the mean values found or the differences between mean values.

The exclusion criteria were: (1) studies where caffeine intake was not associated to VEMP; (2) studies containing subjects with vestibular pathologies, degenerative pathologies of the central nervous system or skeletal muscles and; (3) duplicate publications.

### Data extraction

The titles and abstracts of the retrieved articles were independently assessed by two investigators who were not blinded to the authors or titles of journals. Any disagreements were resolved by consensus. In cases where there was no consensus, a third author was asked to make the final decision. The full versions of potentially eligible articles were retrieved for further evaluation.

The primary outcome assessed in the studies was the VEMP responses without and after caffeine intake by the patients, considering latency for the p13/p1 wave, latency for the n23/n1 wave (both in milliseconds), and p13-n23/p1-n1 amplitude (in microvolts). In addition, as secondary outcomes, the following were assessed: year of study, place of study, age group involved, amount of caffeine intake and time of administration.

All necessary information was extracted from the published papers, protocols and comments related to each study and, where necessary, the authors were contacted for additional information.

In addition to the outcome data, the names of the authors, title, year of publication, country, age ranges of the groups, number of subjects in each group, type and amount of caffeine used were also extracted. A standard form for data storage was created based on the model adopted by Cochrane.^25^

### Bias risk assessment

The risk of bias was assessed according to the recommendations of the “Newcastle-Ottawa” scale and manual, adapted for cross-sectional observational studies. The quality of the studies was independently evaluated by two researchers and the disagreements were evaluated by consensus. The maximum score to be reached was ten points and the evaluated items of the scale were: (1) representativeness of the sample; (2) sample size; (3) management of non-responses; (4) exposure calculation (risk factor); (5) comparability, to investigate whether individuals in different groups of outcomes are comparable, based on study design or analysis, control of confounding factors; (6) evaluation of results and (7) statistical test (Appendix 2).

### Data analysis

The latency results of P13 and N13 were analyzed. For this purpose, a random effects model was used as a measure of the mean difference effect between the groups and as a statistical method of analysis. A value of *α* less than 0.05 was considered statistically significant.

Statistical heterogeneity between the studies was tested using the Cochran test and the inconsistency was tested using the *I*^2^ test. A *p*-value < 0.10 was considered statistically significant. When necessary, study characteristics considered as potential sources of heterogeneity were included in a subgroup analysis. Furthermore, in case of heterogeneity, the studies were removed one by one to investigate whether the removed study was the source of heterogeneity.

All analyses were carried out using RevMan 5.3 software (Cochran Collaboration).

## Results

### Included studies

**Figure 1:**
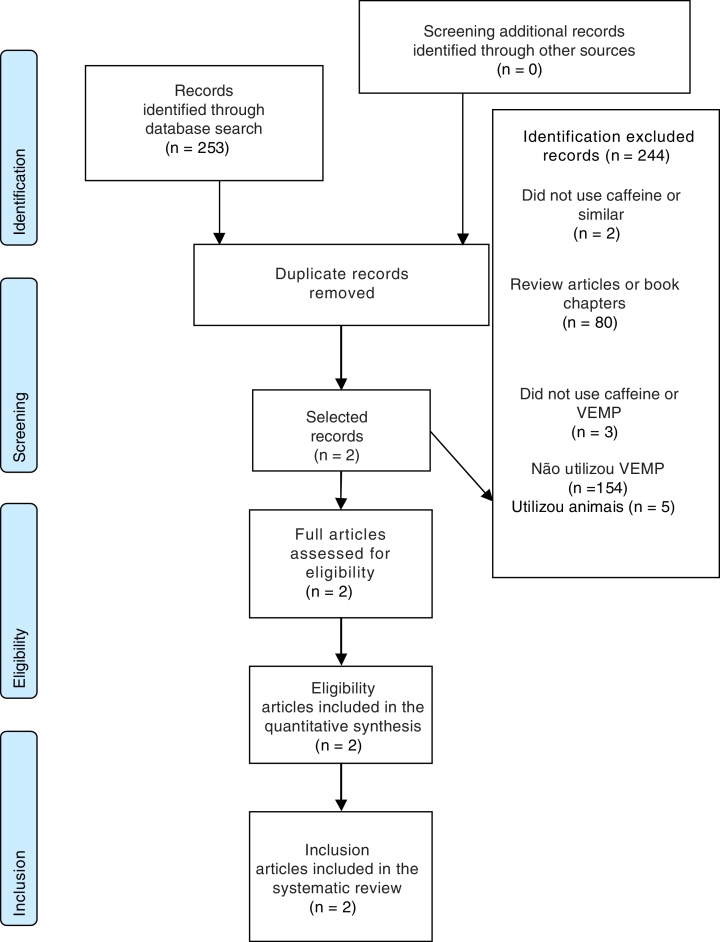
Flowchart diagram of study selection.

Of 253 potentially relevant records identified through the database search, only two full-text publications were retrieved for further evaluation, which were maintained for qualitative analysis (Table 1). The flowchart diagram illustrating the search and selection of the studies is shown in Fig. 1.

**Table 1:** Overall characteristics of included studies.

Source	City (country)	Sample (gender)	Mean age (years) ± standard deviation
McNerney, Coad, Burkard (2014)^26^	New York (USA)	30 patients	23.28 (±1.95)
Sousa, Suzuki (2014)^27^	São Paulo (Brazil)	25 patients	29 years (mean of 25–37 years)

Table 2 shows the characteristics of the performed intervention and the results found by the articles included in this systematic review.

**Table 2:** Characteristics of the included studies regarding the intervention and the results found.

Source	Sessions	Administered amount	Type of supplementation	Examination performed	cVEMP without caffeine administration	cVEMP with caffeine administration
McNerney, Coad, Burkard (2014)^26^	C Session (use of caffeine)	300 mg/individual	Use of coffee with cream or sugar (Starbucks)	cVEMP	P1 (right): 13.62 ± 1.2 P1 (left): 14.13 ± 1.2	P1 (right): 13.46 ± 1.1 P1 (left): 13.94 ± 1.2
	NC Session (no caffeine use)				N1 (right): 20.44 ± 1.6 N1 (left): 20.95 ± 1.4	N1 (right): 20.68 ± 1.8 N1 (left): 20.97 ± 1.8
					Amplitude p1n1 (right): 206.54 ± 123.4	Amplitude p1n1 (right): 187.63 ± 115.6
					Amplitude p1n1 (left): 189.72 ± 122.9	Amplitude p1n1 (left): 194.76 ± 148.6
Sousa, Suzuki (2014)^27^	Two examinations were performed in each of the volunteers, with a mean interval of 60 min.	420 mg/individual	Capsule	cVEMP	P13: 13.41 ± 1.27	P13: 13.56 ± 1.39
					N23: 23.24 ± 2.74	N23: 23.14 ± 2.71
					Amplitude p13n23: 71.90 ± 47.85	Amplitude p13n23: 76.82 ± 48.22

Right P1 and N1 refer to the VEMP record on the right side of the sternocleidomastoid and left P1 and N1 to its left side.

Considering the recommendation of abstaining from the consumption of foods and beverages containing caffeine 24 h before undergoing vestibular tests, McNerney et al. (2014)^26^ sought to investigate whether and how caffeine influences the outcome of vestibular caloric tests and cVEMP, common clinical tests for the analysis of vestibular function. For that purpose, they assessed a sample of 30 healthy young adults, of which 21 were females, with a mean age of 23.28 ± 1.95 years. This study reported the occurrence of two sessions during the study performance, with session C being the one in which the individuals received caffeine in coffee (the equivalent of 300 mg of caffeine) before the vestibular function tests, allowing the addition of cream or sugar at the volunteer's discretion. The NC session was referred to as the one in which individuals did not consume caffeine, which was not permitted within 24 h before the test was performed. Both sessions were counterbalanced. Moreover, a caffeine withdrawal scale was applied, which included a series of symptoms resulting from this withdrawal (headache, fatigue, nausea, lack of concentration among others), where 0 meant the absence of symptoms and 10, the presence of severe symptoms. Additionally, volunteers described the amount and frequency of consumption of caffeine-rich foods/beverages for an estimated calculation of monthly consumption.^26^

After the tympanometry, the vestibular clinical tests were performed, and the Variotherm Plus Caloric Irrigator was used in the vestibular caloric tests to administer the water, while the software l-Portal VNG was used to collect and analyze the subjects’ eye movements. The TECA evoked potential system was used to obtain cVEMP. The tone burst stimulus was given at 500 Hz frequency and was presented at a rate of approximately 6 Hz. Each cVEMP measurement was pro-mediated by 100 stimuli, with the recovery time being approximately one minute after each data acquisition period, aiming to reduce the effects of muscle fatigue. The subjects were asked to produce muscular contraction of the sternocleidomastoid by lifting their heads from a supine position and away from the stimulated ear.^26^

The results showed that the intake of a moderate amount of caffeine did not substantially influence the clinical interpretation of any of the tests. Moreover, the results of the caffeine withdrawal questionnaire indicated that abrupt cessation of caffeine intake may result in withdrawal symptoms, such as headache, nausea, fatigue, and anxiety.^26^

The study by Souza and Suzuki (2014),^27^ aimed at assessing the interference of the acute use of caffeine in the vestibulo-collic reflex through cVEMP. Twenty-five healthy young adult subjects were selected, 68% females and 32% males, with a mean age of 29 years (25–37 years), in a convenience sample. These subjects were instructed to abstain from caffeine for at least 24 h before the vestibular test, and after VEMP was performed, two capsules with pure caffeine (210 mg/capsule) were provided with 100 mL of filtered water for each of volunteers. After a mean interval of 60 min, the second examination was performed.

Each volunteer was placed in a standardized position, so that the sternocleidomastoid muscle was contracted at the time of recording. The rarefied tone burst stimulus was given at an intensity of 100 decibels of a sound pressure level and a frequency of 1000 Hz. The presentation rate was 5 Hz. Two binaural measurements were performed, with a 2-minute interval between them for patient rest, and 200 stimuli were pro-mediated in each of these measurements. There was no statistically significant difference between the examinations before and after the drug use.^27^

The two included articles used the same group of volunteers to constitute the control group and the intervention group of the study. VEMP was initially performed without the previous administration of caffeine and, in a second moment, after caffeine administration. The amount/dose and the type of caffeine used differed between the two studies. Both studies chose to analyze the Cervical Vestibular Evoked Myogenic Potential (c-VEMP), but the methodology used by the authors differed. The latency of P13/P1 and N23/N1, as well as the amplitude of the P13-N23/P1N1 response were recorded and analyzed for each subject of the abovementioned studies.

### Bias risk assessment

The analysis of the quality of the included articles and, consequently, the risk of bias, is shown in Table 3. The two included studies^26^, ^27^ are characterized as observational and cross-sectional studies. Additionally, in the final evaluation, they obtained a percentage of quality equal to 70% (7/10) and 80% (8/10).

**Table 3:** Quality of the articles included, according to the Newcastle–Ottawa Quality Assessment Scale.

Authors	Sample representativeness	Justified sample size^a^	Rate of non-responses	Exposure assessment	Comparability	Assessment of results	Appropriate statistical test	Final assessment^b^
McNerney, Coad, Burkard (2014)^26^	Non-representative (0)	Yes (1)	0% (1)	Validated tool (2)	Yes (2)	Study's own report (1)	Yes (1)	8/10
Sousa, Suzuki (2014)^27^	Non-representative (0)	No (0)	0% (1)	Validated tool (2)	Yes (2)	Study's own report (1)	Yes (1)	7/10

a Minimum criterion of *n* ≥ 30 (central limit theorem).

b Maximum score of 10 stars. Result presented as: points obtained/maximum score.

Neither study was concerned with the sample representativeness, that is, they chose the groups for convenience. The size of the adult sample of one of the studies^26^ was satisfactory, as it fit the central limit theorem, with samples larger than 30 subjects. However, neither of them performed calculations to estimate their sample size.

The non-response rate was satisfactory in both studies. The studies^26^, ^27^ used validated tools for data collection and the comparability between the control group and the group that received the caffeine was also possible for all of them. The evaluation of results was carried out in all studies by means of their own report. Finally, all studies used appropriate statistical tests.

### Data analysis

**Figure 2:**
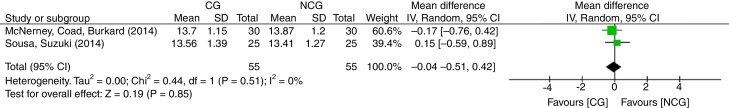
Global effect of caffeine on P13 latency.

**Figure 3:**
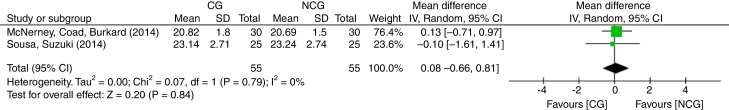
Global effect of caffeine on N23 latency.

Considering the objectives and methodologies of the selected articles, quantitative analyses of the data that showed results in common were performed. Thus, the meta-analysis was performed for P13 and N13 latencies and are shown in Figure 2, Figure 3, respectively.

The use of caffeine had a greater influence on P13 latency (RR: -0.04 [0.51-0.42], *p* = 0.85); however, no statistically significant differences were found between groups.

Non-use of caffeine had a greater influence on N23 latency (RR: 0.08 [−0.66 to 0.81], *p* = 0.84), but no statistically significant differences were found between the groups.

Significant differences were not observed for the amplitudes, either; however, it was not possible to perform the meta-analysis as it was done for the latency parameter, since the study by Sousa and Suzuki (2014)^27^ did not show the data per separate components (P13 and N23), but rather the P13-N23 inter-amplitude.

## Discussion

The methodological quality of the studies was satisfactory, reaching at least 70% of the maximum score. The fact that the studies^26^, ^27^ used convenience sampling is a matter of concern, and very common in scientific studies, as they do not allow the composition of representative samples. On the other hand, they used validated tools for data collection and appropriate statistical tests, which shows a greater concern with the quality of the quantitative analyses of these studies.

The studies show that caffeine does not alter the response patterns in cVEMP in normal subjects. Considering this result, it is necessary to investigate and observe whether such lack of effect on cVEMP also occurs in patients with labyrinthine disease. Abrupt and total restriction of caffeine in preparation for neurotological examinations generates inconvenient symptoms such as headaches, nausea, anxiety, and withdrawal irritability. Thus, if there is no effect of caffeine on cVEMP responses in patients undergoing neurotological investigation, the abovementioned inconveniences can be avoided by maintaining caffeine consumption prior to the examination. Considering the limitations shown here and especially the absence of randomized clinical trials on this subject, generalizations cannot be made.

The importance of carrying out other studies to analyze whether these same results are applicable to older adults and individuals with impaired vestibular function are verified, as well as whether the chronic use of caffeine and VEMP acquired from other muscles do not affect the results.

## Conclusion

Considering the articles evaluated here, there is no evidence that caffeine influences vestibular evoked myogenic potential in normal subjects, and more studies with adequate methodologies are necessary.

## Conflicts of interest

The authors declare no conflicts of interest.

## Figures and Tables

**Table:** 

*MEDLINE (via PubMed)*
#1 (“caffeine”[MeSH Terms] OR Caffeine [Text Word] OR “coffee”[MeSH Terms] OR coffee [Text Word])
#2 “vestibular evoked myogenic potentials”[MeSH Terms] OR Ocular Vestibular Evoked Myogenic Potentials [Text Word] OR “vestibular evoked myogenic potentials”[MeSH Terms] OR cervical vestibular evoked myogenic potentials [Text Word]
#4 #1 AND #2

*Cochrane Clinical Trials*
(caffeine OR coffee) AND (“vestibular evoked myogenic potentials” OR “Ocular Vestibular Evoked Myogenic Potentials” OR “cervical vestibular evoked myogenic potentials”): ti,ab,kw

*ScienceDirect*
ALL ((caffeine OR coffee)) and ALL((“vestibular evoked myogenic potentials” OR “Ocular Vestibular Evoked Myogenic Potentials” OR “cervical vestibular evoked myogenic potentials”))

*ClinicalTrials.gov*
caffeine OR coffee AND “vestibular evoked myogenic potentials” OR “Ocular Vestibular Evoked Myogenic Potentials” OR “cervical vestibular evoked myogenic potentials” | Closed Studies | Studies With Results | Interventional Studies

*LiLACS*
cafeína and “potencial evocado miogênico vestibular”

*Scopus*
TITLE-ABS-KEY((caffeine OR coffee) AND (“vestibular evoked myogenic potentials” OR “Ocular Vestibular Evoked Myogenic Potentials” OR “cervical vestibular evoked myogenic potentials”))

*Web of Science*
Topic:(caffeine OR coffee) AND Topic: (“vestibular evoked myogenic potentials” OR “Ocular Vestibular Evoked Myogenic Potentials” OR “cervical vestibular evoked myogenic potentials”)
Circumpolar Health Bibliographic Database; The New York Academy of Medicine, ClinicalEvidence.com
caffeine OR coffee AND “vestibular evoked myogenic potentials” OR “Ocular Vestibular Evoked Myogenic Potentials” OR “cervical vestibular evoked myogenic potentials”

*Scielo*
caffeine AND “vestibular evoked myogenic potentials”

*OPENGREY.EU*
caffeine OR coffee AND “vestibular evoked myogenic potentials” OR “Ocular Vestibular Evoked Myogenic Potentials” OR “cervical vestibular evoked myogenic potentials”

*DissOnline.de*
“caféine” AND “vestibulaires potentiel évoqué myogénique”

**Table:** 

**Selection: (Maximum of 5 stars)**
*1) Sample representativeness:*
a) Truly representative of the mean in the target population.* (All subjects or random sampling)
* *b) Slightly representative of the mean in the target population.* (Non-random sampling)
c) Selected group of users.
d) Description of the sampling strategy.
*2) Sample size:*
a) Justified and satisfactory.*
b) Not justified.
*3) No answers:*
a) The comparability between responses and non-responses is established – and the response rate is satisfactory.*
b) The response rate is not satisfactory, or the comparability between responses and non-respondents is unsatisfactory.
c) Description of response rate or characteristics of responses and non-responses.
*4) Exposure calculation (risk factor):*
a) Validated measurement tool.**
b) The measuring tool is not validated, but the tool is available or described.*
c) Description of the measuring tool.

**Comparability: (Maximum of 2 stars)**
*1) The objects in different result groups are comparable, based on the study design or analysis. Confounding factors are controlled*.
a) The study takes into account the most important factor (select one). *
b) Study control for any additional factor.*

**Result: (Maximum of 3 stars)**
*1) Evaluation of results:*
a) independent blind evaluation.**
b) Record association.**
c) Study's own report.*
d) no description.
*2) Statistical test:*
a) The statistical test used to analyze the data are clearly described and adequate, and association measurement is shown, including confidence intervals and the level of probability (*p*-value).*
b) The statistical test is not appropriate, not described or incomplete.
*This scale was adapted from the Newcastle–Ottawa Quality Assessment Scale for cohort studies to perform an evaluation of cross-sectional quality studies for the systematic review, “Are healthcare workers’ intentions to vaccinate related to their knowledge, beliefs and attitudes? A systematic review”.*
